# International Airport Wastewater as a Sentinel Site for Genomic Surveillance of Human Viruses and Bacteriophages

**DOI:** 10.3390/microorganisms14071402

**Published:** 2026-06-25

**Authors:** Ana Paula Assad de Carvalho, Mariana Silva Almada, Cíntia Dutra Leal, Josiane Fernandes, Maria Cristina Costa, Vagner de Souza Fonseca, Marta Giovanetti, Luiz Carlos Junior Alcantara, Juliana Calábria de Araújo

**Affiliations:** 1 Department of Sanitary and Environmental Engineering, School of Engineering, Universidade Federal de Minas Gerais (UFMG), Belo Horizonte 31270-901, MG, Brazil; assadanapaula@gmail.com (A.P.A.d.C.); cintia@desa.ufmg.br (C.D.L.); engjsfernandes@gmail.com (J.F.); 2 Centro Federal de Educação Tecnológica de Minas Gerais (CEFET-MG), Belo Horizonte 30421-169, MG, Brazil; marisilvalmada@gmail.com (M.S.A.); cristinacosta.cefetmg@gmail.com (M.C.C.); 3 Department of Exact and Earth Sciences, Universidade do Estado da Bahia, Salvador 41150-000, BA, Brazil; vagner.fonseca@gmail.com; 4 Centre for Epidemic Response and Innovation (CERI), School of Data Science and Computational Thinking, Stellenbosch University, Stellenbosch 7600, South Africa; 5 Department of Sciences and Technologies for Sustainable Development and One Health, Università Campus Bio-Medico di Roma, 00128 Rome, Italy; giovanetti.marta@gmail.com; 6 René Rachou Institute, Fundação Oswaldo Cruz (FIOCRUZ MINAS), Belo Horizonte 30190-001, MG, Brazil

**Keywords:** airport wastewater, virome, viral pathogens, multipathogen monitoring, bacteriophages, wastewater surveillance, environmental surveillance, genomic surveillance, travel-associated surveillance, hybrid-capture sequencing

## Abstract

Airports are strategic targets for wastewater-based epidemiology because they concentrate highly mobile populations and may provide early signals of pathogen circulation. However, metagenomic investigations of airport wastewater remain limited, particularly in South America. Here, we present one of the first hybrid-capture target-enriched metagenomic investigations of airport wastewater in Brazil, integrating the detection of human-associated viruses and bacteriophage-derived host signatures to evaluate airports as sentinel surveillance sites. Seven untreated wastewater samples collected from a major Brazilian airport between December 2021 and March 2023 were concentrated, subjected to nucleic acid extraction, and analyzed using hybrid-capture target-enriched next-generation sequencing. Taxonomic analysis identified 615 viral and bacteriophage-associated taxa, including 440 viruses and 175 bacteriophages. Among the viral fraction, 21 human-associated viral taxa representing eight viral families were selected for detailed analysis. *Norovirus GII* was detected in all samples, while *Mamastrovirus 1* and *JC polyomavirus* were detected in six of seven samples. SARS-CoV-2 and dengue virus type 1 were simultaneously detected in the March, 2023 sample. The bacteriophage fraction comprised 47 host-associated phage groups, with *Streptococcus*-associated phages predominating across samples. These findings demonstrate that airport wastewater can capture diverse human viral and bacteriophage-derived signatures associated with population mobility, supporting its application in environmental genomic surveillance and early-warning systems for emerging and circulating pathogens.

## 1. Introduction

Airports are strategic interfaces between local populations and global mobility, concentrating large and constantly changing groups of travelers, workers, and service activities. This intense circulation can facilitate the rapid dissemination of infectious agents across regions and countries, while also making airports valuable sites for wastewater-based epidemiology (WBE). Wastewater surveillance can capture biological signals from infected individuals and complement conventional clinical surveillance [1]. In airport settings, wastewater monitoring has been shown to support public health surveillance by detecting SARS-CoV-2 and monitoring viral circulation in highly connected environments [2,3,4,5,6]. The relevance of this approach became particularly evident during the COVID-19 pandemic, when wastewater-based surveillance was increasingly recognized as a tool for early warning and border-related monitoring.

Since then, airport and aviation-associated wastewater surveillance has expanded beyond SARS-CoV-2. Airport sewage has been used to investigate poliovirus importation, and mpox virus DNA has also been detected in wastewater, including airport-associated samples [7,8,9]. More recent studies have shown that aircraft and airport wastewater can be useful for monitoring multiple pathogens, including SARS-CoV-2 variants and other viral targets [10,11]. In parallel, aircraft wastewater collected from arriving flights has been explored as a more travel-specific surveillance source. Metagenomic analyses of toilet waste from long-distance flights have revealed a broad diversity of viruses, and aircraft wastewater has been used to detect SARS-CoV-2, variants of concern, and enteric and respiratory viruses [12,13,14,15,16]. Additional studies have highlighted the usefulness of aircraft wastewater for genomic and pathogen surveillance in travel-associated contexts [17,18,19,20]. Although both wastewater sources are relevant, airport wastewater treatment plant influent and aircraft lavatory wastewater represent different surveillance scales: aircraft wastewater may capture more flight-specific signals, whereas airport wastewater integrates contributions from passengers, workers, commercial areas, sanitary facilities, food services, and other airport operations.

Despite these advances, most airport and aircraft wastewater studies have focused on single pathogens, SARS-CoV-2 variants, or specific public health events. Broader investigations of the viral composition of airport wastewater remain limited, and bacteriophage communities are still rarely explored in this context. Although previous studies have demonstrated the usefulness of airport wastewater for detecting selected viral targets, there remains a need for approaches that provide a broader overview of viral and bacteriophage-associated signatures in untreated airport wastewater. By applying hybrid-capture target-enriched sequencing, such investigations can simultaneously characterize multiple human-associated viruses and bacteriophage-derived host signatures, offering insights into viral diversity, human-associated microbial inputs, and pathogen signals potentially circulating in highly mobile and mixed populations. This is particularly relevant in tropical and subtropical regions such as Brazil, where enteric, respiratory, urinary-associated, and arboviral pathogens may circulate simultaneously and are not fully captured by single-target monitoring approaches.

In this context, the present study aimed to characterize the viral and bacteriophage composition of airport wastewater from a major Brazilian airport using a hybrid-capture target-enriched sequencing approach. By integrating human-associated viral detection with bacteriophage-derived read profiling, this study evaluates the potential of airport wastewater as a strategic source of information for multipathogen surveillance, early warning, and ecological interpretation of microbial signals in a high-mobility environment.

## 2. Materials and Methods

### 2.1. Characterization of the Sampling Site

The airport investigated is located 41 km from the center of Belo Horizonte, the capital of Minas Gerais, Brazil [21]. The metropolitan region of Belo Horizonte has a population of approximately 5.73 million inhabitants, making it the third most populous in the country [22]. Covering an area of 132,000 m^2^ and handling approximately 198,000 annual aircraft movements, the airport serves around 70 domestic and international destinations, making it the third-largest airport in the country in terms of route network [23]. In 2022 and 2023, it ranked as the fifth-busiest airport in the country by passenger traffic, with 9.2 million and 10.0 million passengers, respectively. By 2025, this figure had increased to 12.1 million passengers [24]. During the studied period, the airport functioned as an important domestic air hub, serving multiple Brazilian destinations. Its regular direct international routes were limited to Lisbon, Portugal, and Panama City, Panama, until late March 2023, when a direct connection with Bogotá, Colombia, was added [25]. By May 2026, at the time of manuscript submission, the airport’s direct international network had expanded to include Lisbon, Orlando, Panama City, Santiago, Buenos Aires, and Montevideo [26].

Seven wastewater sampling campaigns were carried out at the airport on 29 December 2021; 26 January, 10 February, 27 April, 18 May and 1 June 2022 and 15 March 2023. Samples were collected at the Airport Wastewater Treatment Plant (WWTP) before treatment, where all effluents generated at the passenger terminal, cargo terminal, and administrative buildings are conveyed, including wastewater from sanitary facilities and kitchens. In addition, WWTP receives domestic sewage and post-effluent from the preliminary treatment system installed in the maintenance hangar of one of the airlines [27]. The sampling dates were defined according to the operational schedule and sample collection availability within the wastewater surveillance project and were not selected to coincide with specific peak travel periods or airport traffic events. No wastewater sample was collected in March 2022; therefore, a sample from that month was not included in the analysis.

**Figure 1:**
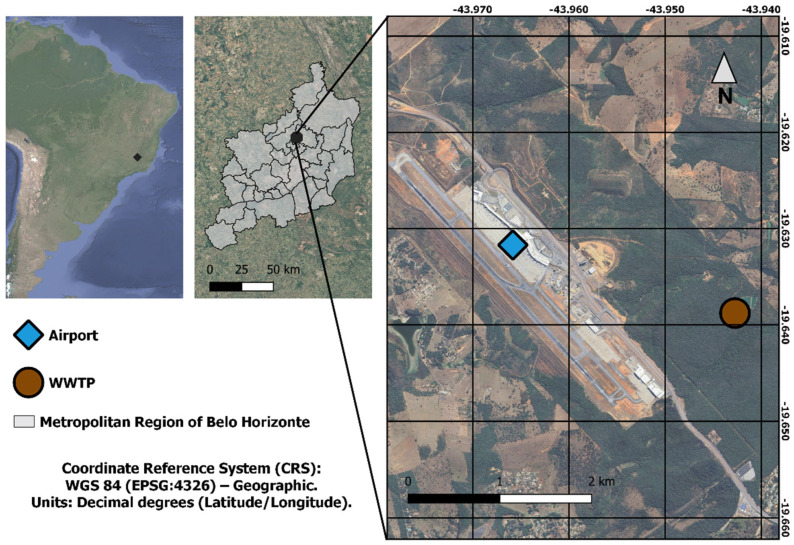
Map of the location of the investigated airport and the site for collecting wastewater samples from the airport (WWTP).

The locations of the investigated airport and its WWTP are shown in Figure 1. Previous long-term characterization of these wastewater samples showed that, overall, the airport effluent presented physicochemical and bacteriological parameter concentrations similar to those typically found in domestic sewage, indicating the characteristics of more diluted sewage [27]. Supplementary Table S1 summarizes the median values of selected parameters analyzed over 17 years of monitoring.

### 2.2. Sample Processing and Extraction of Genetic Material

All samples were collected as 8-h flow-proportional composite samples during weekday daytime hours within the operational sampling window of 7:00 a.m. to 7:00 p.m. Composite samples were prepared from hourly aliquots, the volume of each aliquot being adjusted according to the hourly influent flow profile of the airport WWTP, using the reference flow pattern established from the previous month’s data. Samples were maintained under refrigerated conditions during transport to the laboratory. Samples were processed within 24 h of collection. Briefly, 30 to 50 mL of each homogenized wastewater sample was filtered through 47 mm mixed cellulose ester membranes with a pore size of 0.45 μm, following a previously described protocol [28]. After filtration, each membrane was transferred to a sterile 2 mL microtube and stored at −80 °C until nucleic acid extraction. Total nucleic acids were extracted using the commercial AllPrep^®^ PowerViral^®^ DNA/RNA Kit (Qiagen^®^, Hilden, Germany), following the manufacturer’s instructions. Extracted nucleic acids were eluted in 100 microliters, assessed for concentration and purity using a NanoDrop™ Lite spectrophotometer (Thermo Scientific^®,^ Waltham, MA, USA), and stored at −80 °C until library preparation.

### 2.3. Hybrid-Capture Target Enrichment for Viral Pathogens and Bacteriophage-Derived Reads

Target enrichment was conducted using a hybrid-capture approach. Sequencing libraries were prepared from extracted total nucleic acids using the Illumina RNA Prep with Enrichment kit and Illumina RNA Prep with Enrichment Indexes Set A, following the manufacturer’s protocol. Target enrichment was performed using the Illumina Viral Surveillance Panel (VSP; Illumina, San Diego, CA, USA), a hybrid-capture oligonucleotide probe panel designed for broad viral surveillance of selected DNA and RNA viruses. Because this workflow is based on probe hybridization and capture rather than amplicon sequencing, no virus-specific PCR primers were used.

Briefly, indexed libraries were generated, pooled, hybridized with the VSP probe panel, captured using streptavidin magnetic beads, amplified, purified, and quantified before sequencing. Enriched libraries were sequenced on the Illumina NextSeq™ 2000 platform (San Diego, CA, USA) using a paired-end 2 × 150 bp configuration [29]. For VSP-enriched libraries, the manufacturer recommends a minimum sequencing depth of 2 million paired-end reads for low-complexity samples and 8 million paired-end reads for high-complexity samples, including wastewater. In the present study, the total number of read pairs generated per sample ranged from 12,620,114 (18 May 2022) to 27,406,172 (27 April 2022), exceeding the recommended minimum depth for complex wastewater samples.

Sequence quality was assessed using FastQC, and reads were trimmed using Trimmomatic. After quality control, between 92.9% and 96.1% of reads were retained per sample. Following viral enrichment filtering, between 502,210 and 1,455,918 reads per sample passed all filters and were used for downstream taxonomic classification and assembly. The number of reads mapped back to assembled contigs ranged from 15,980 (15 March 2023) to 291,487 (1 June 2022) per sample, reflecting variation in viral load, target enrichment efficiency, and overall viral diversity across sampling dates. These sequencing and processing statistics are reported in Supplementary Table S2.

The bioinformatic workflow included viral read classification and filtering with DIAMOND and Kraken, de novo assembly with (Meta-)SPAdes, scaffolding against reference genomes, and consensus sequence generation with BCFTools or GATK for highly similar strains; for more divergent strains, polished draft genomes were generated. Viral reads and bacteriophage-derived reads recovered from the raw sequencing data were retained for downstream analysis. Bacteriophage-derived reads were subsequently grouped according to their annotated bacterial hosts, allowing for the characterization of host-associated phage profiles across samples. Abundance was calculated as the percentage of reads assigned to each viral or bacteriophage taxon relative to the total number of assigned reads in each sample, and values were then log10-transformed for visualization. Findings related to selected human viruses, including mpox virus (MPXV), dengue virus (DENV), chikungunya virus (CHIKV), and Zika virus (ZIKV), have been described elsewhere [28,30].

## 3. Results and Discussion

### 3.1. Human-Associated Viral Profiles in Airport Wastewater

**Figure 2:**
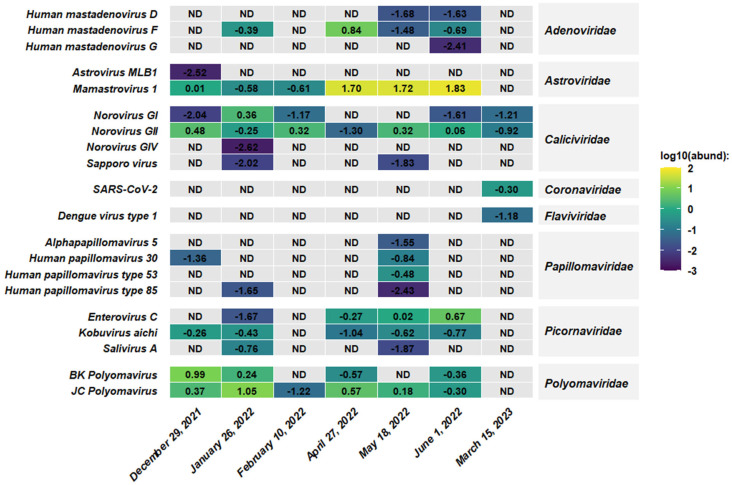
Temporal profile of human-associated viral taxa detected in airport wastewater samples. ND: not detected. Abundance values represent the log10-transformed percentage of reads assigned to each viral taxon relative to the total number of viral reads assigned in each sample.

Hybrid-capture target-enriched NGS detected taxonomic assignments to 615 viral and bacteriophage-associated taxa across the airport wastewater samples, comprising 440 viruses and 175 bacteriophages. From the viral fraction, 21 human-associated viral taxa of public health relevance were selected for detailed analysis (Figure 2). The number of viral reads considered in the analysis varied among samples: 66,463 (29 December 2021), 84,178 (26 January 2022), 61,755 (10 February 2022), 184,735 (27 April 2022), 161,545 (18 May 2022), 284,293 (1 June 2022), and 51,430 (15 March 2023).

The selected viral taxa were assigned to eight families: *Adenoviridae*, *Astroviridae*, *Caliciviridae*, *Coronaviridae*, *Flaviviridae*, *Papillomaviridae*, *Picornaviridae*, and *Polyomaviridae*. The detected profile included human *mastadenovirus D*, *F*, and *G*; *Astrovirus MLB1* and *Mamastrovirus 1*; *Norovirus GI*, *GII*, and *GIV; Sapporo* virus; SARS-CoV-2; dengue virus type 1; *Alphapapillomavirus 5*; human papillomaviruses 30, 53, and 85; *Enterovirus C*; *Kobuvirus aichi*; *Salivirus A*; and *BK* and *JC polyomaviruses*. Overall, these findings show that wastewater from a major Brazilian airport can capture a broad spectrum of human-associated viral signals, including enteric, urinary-associated, respiratory, and arboviral targets. Previous airport wastewater studies demonstrated the value of this approach for monitoring SARS-CoV-2 and its variants, and airport and aircraft wastewater have also been shown to be useful for multipathogen surveillance [2,3,5,6,10]. Together, these studies support the use of airport wastewater as a complementary source of surveillance information in highly connected environments, particularly when monitoring is expanded beyond single-pathogen approaches.

Detection frequency varied substantially among viral taxa and sampling dates. *Norovirus* GII (genogroup II) was detected in all seven samples, indicating a persistent enteric viral signal throughout the study period. *Mamastrovirus 1* and *JC polyomavirus* were detected in six of seven samples, whereas *Norovirus* GI and *Kobuvirus aichi* were detected in five samples. *Human mastadenovirus* F, *Enterovirus* C, and *BK polyomavirus* were each detected in four samples. Other taxa showed sporadic detection, including *Astrovirus* MLB1, *Norovirus* GIV, SARS-CoV-2, dengue virus type 1, *Alphapapillomavirus 5*, *human papillomavirus* type 53, and *human mastadenovirus* G, which were each detected in only one sample. The number of detected viral taxa per sample ranged from four to thirteen, with the lowest richness observed on 10 February 2022 and the highest observed on 18 May 2022.

The recurrent detection of enteric and human-associated viruses was a prominent feature of the dataset. The detection of *Norovirus* GII, *Norovirus* GI, *Mamastrovirus* 1, *Kobuvirus aichi*, *Enterovirus* C, and *Salivirus* A indicates that sequencing of airport wastewater repeatedly captured viral signals associated with gastrointestinal shedding and fecal inputs. This interpretation is consistent with diarrheal disease surveillance data from Minas Gerais, where more than 500,000 cases of diarrhea were reported in 2022, and 227,845 cases and 264 outbreaks had been reported in 2023 by July, although publicly available data does not provide systematic etiological confirmation for these events [31]. At the national level, *norovirus* and *rotavirus* were among the etiological agents identified in reported outbreaks of waterborne and foodborne diseases in Brazil from 2014 to 2023 [32]. In addition, a large acute gastroenteritis outbreak affecting several municipalities in Santa Catarina during the 2023 summer season was associated with multiple norovirus genotypes after storm events [33].

These epidemiological data support the interpretation that recurrent detection of noroviruses and other enteric viruses in airport wastewater is consistent with the broader circulation of gastrointestinal viruses in Brazil, while direct comparison with local clinical cases remains limited by the lack of systematic etiological confirmation. Wastewater monitoring has been shown to be applicable for *Norovirus GII* surveillance, and the relevance of noroviruses and other enteric viruses in wastewater surveillance has also been reinforced [34,35,36]. Similarly, studies of aircraft lavatory wastewater and toilet waste from long-distance flights have demonstrated the detection of indicator, enteric, and respiratory viruses [12,16]. The detection of human adenoviruses and BK and *JC polyomaviruses* further support the human-associated origin of the wastewater signal, since human adenoviruses have been described as relevant waterborne index pathogens and human polyomaviruses, particularly JC and BK viruses, have been proposed as markers of human fecal or sewage contamination [37,38].

Because samples were collected from the airport WWTP influent, the viral profile should be interpreted as an integrated signal from airport activities rather than as a passenger-specific signal. The airport WWTP receives effluents from multiple sources, including passenger terminals, administrative buildings, sanitary facilities, kitchens, and aircraft maintenance-related wastewater [27]. This mixed wastewater stream likely reflects contributions from travelers, airport workers, food services, sanitary facilities, and other operational areas. Similar interpretations have been reported in airport and aviation-associated wastewater studies, in which wastewater signals are understood as composite indicators influenced by population mobility, infrastructure, and sampling context [3,10]. Therefore, recurrent detection of enteric and urinary-associated viruses may indicate a stable human-associated wastewater background in the airport environment, while sporadic detections may reflect temporal changes in pathogen circulation, wastewater inputs, or sequencing recovery.

Log10-transformed relative read abundance values also varied across taxa and sampling dates. These values represent the log10 of the percentage of reads assigned to each viral taxon relative to the total number of viral reads assigned in each sample. *Mamastrovirus 1* showed the highest relative read abundance among the selected viruses, reaching log10(abundance) values of 1.70, 1.72, and 1.83 in the samples collected on 27 April, 18 May, and 1 June 2022, respectively. *JC polyomavirus* and *BK polyomavirus* were also recurrent, with maximum log10(abundance) values of 1.05 and 0.99, respectively. However, these values should be interpreted as relative read-based measures rather than absolute viral loads, since sequencing-derived abundance estimates may be influenced by sample concentration, nucleic acid recovery, target enrichment efficiency, sequencing depth, and bioinformatic classification. Sequencing-based relative abundance estimates can be affected by systematic experimental biases, reinforcing the need for cautious interpretation of read-based abundance values [39].

The sample collected on 15 March 2023 showed a distinct viral profile, with the detection of SARS-CoV-2 and dengue virus type 1, which were not detected in the previous samples analyzed in this study. Dengue virus type 1 was detected during a period of increased dengue transmission in Brazil and Minas Gerais. National epidemiological data showed that the dengue incidence in Brazil exceeded the upper limit of the endemic channel between epidemiological weeks 9 and 22 of 2023, a period that includes the March 2023 sampling date [40]. In Minas Gerais, dengue activity was also substantial in 2023, with 327,238 reported cases and 204 deaths from January to December [41]. In contrast, SARS-CoV-2 was only detected in the 15 March 2023 sample, despite municipal surveillance data showing substantial COVID-19 activity in Belo Horizonte during the broader study period, especially in 2021 and 2022 [42]. In the epidemiological bulletin published on the sampling date, Belo Horizonte had recorded 200,978 confirmed COVID-19 cases in 2021, 159,362 in 2022, and 1602 in 2023 up to mid-March [42]. State-level surveillance data from Minas Gerais also showed marked temporal variation in reported COVID-19 cases between 2020 and 2023 [43]. This discrepancy reinforces that airport wastewater should be interpreted as a point-in-time signal from a highly dynamic and mixed population rather than as a direct proxy for infection incidence in the surrounding municipality.

Overall, the viral composition detected in airport wastewater revealed a diverse and dynamic profile, comprising persistent enteric and human-associated signals as well as sporadic detections of viruses with different transmission routes. Previous studies have shown that airport and aircraft wastewater can complement conventional surveillance by capturing viral circulation and pathogen introduction events in settings of intense mobility [2,3,5,6,10]. In this context, the present findings support airport wastewater as a complementary source of information for multipathogen surveillance, particularly for capturing both continuously circulating human-associated viruses and episodic signals from pathogens of public health relevance.

### 3.2. Bacteriophage-Derived Read Profiles in Airport Wastewater

**Figure 3:**
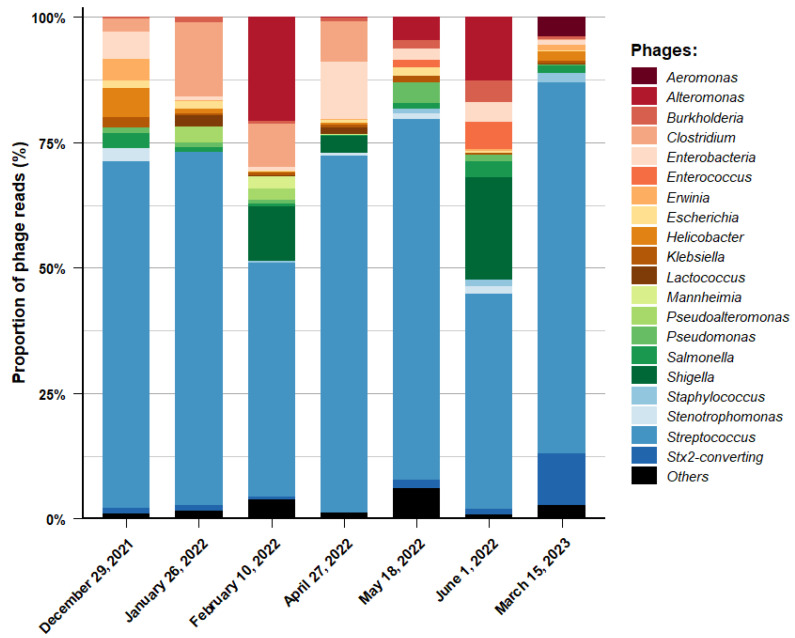
Temporal profile of the bacteriophage community detected in airport wastewater samples. Values represent the proportion (%) of phage reads assigned to each host-associated phage group relative to the total number of phage reads in each sample. The 20 most abundant phage groups are shown individually, while the remaining groups are combined as “Others”.

Phage-derived reads assigned to 175 bacteriophage taxa were detected in airport wastewater samples and grouped into 47 host-associated phage groups based on bacterial host annotation. The 20 most abundant groups are shown in Figure 3, and the complete list is provided in Supplementary Table S3. The number of phage reads varied among samples, with 1971 (29 December 2021), 6734 (26 January 2022), 5525 (10 February 2022), 19,323 (27 April 2022), 5802 (18 May 2022), 3673 (1 June 2022), and 3632 (15 March 2023) reads recovered.

The number of host-associated phage groups detected varied across the sampling period, ranging from 14 groups on 29 December 2021 to 24 groups on 10 February 2022, 27 April 2022, 18 May 2022, and 15 March 2023. Intermediate richness was observed on 26 January 2022, with 22 groups, and on 1 June 2022, with 21 groups. Across all sampling dates, *Streptococcus*-associated phages represented the dominant fraction of the phage profile, accounting for 42.88% to 73.82% of total phage reads. Bacteriophages are abundant components of sewage microbial communities, and phages are closely related to their bacterial hosts in wastewater treatment systems [44,45]. In this context, the persistent predominance of *Streptococcus*-associated phages suggests that this group formed a stable component of the sewage-associated phage background during the study period.

Although *Streptococcus*-associated phages dominated all samples, the relative contribution of secondary phage groups varied substantially over time. *Alteromonas* phages were particularly abundant on 10 February 2022 and 1 June 2022, representing 20.65% and 12.63% of phage reads, respectively. *Shigella* phages also increased on these same dates, reaching 10.84% and 20.34%, while *Clostridium* phages were most abundant on 26 January 2022 and 10 February 2022, with 14.82% and 8.67%, respectively. *Enterobacteria* phages reached their highest proportion on 27 April 2022, accounting for 11.31% of phage reads, whereas Stx2-converting phages were most prominent on 15 March 2023, reaching 10.38%. These temporal shifts indicate that, beyond the persistent *Streptococcus*-associated phage signal, the airport wastewater phage profile was dynamic and may reflect changes in the relative contribution of different host-associated groups. This interpretation is consistent with previous descriptions of phage-bacteria interactions as important drivers of microbial community dynamics and with the recognized role of wastewater phage populations as part of complex microbial communities [44,45].

Several phage groups were recurrent across the sampling period. *Burkholderia*-, *Enterobacteria*-, *Escherichia*-, *Klebsiella*-, *Pseudomonas*-, *Salmonella*-, *Streptococcus*-, and *Stx2*-converting phages were detected in all seven samples, while *Erwinia* phages were detected in six of seven samples. In contrast, *Achromobacter*-, *Aggregatibacter*-, *Arthrobacter*-, *Bacillus*-, *Bacteroides*-, *Escherichia, Stx2 converting*-, *Lactobacillus*-, *Microbacterium*-, *Ochrobactrum*-, *Psychrobacter*-, *Rhizobium*-, and *Rhodoferax* phages were only detected in one sample. This combination of recurrent and sporadic phage groups suggests a stable sewage-associated phage background, accompanied by temporal fluctuations that may reflect changes in wastewater inputs, bacterial host availability, or sequencing recovery. Importantly, the detection of host-associated phage groups should not be interpreted as direct evidence of viable pathogenic bacteria, antimicrobial resistance, or increased bacterial risk in airport wastewater. Rather, these phage profiles provide indirect ecological signatures of bacterial communities commonly associated with human sewage, environmental inputs, and wastewater systems. The recurrent detection of phages associated with genera such as *Escherichia*, *Klebsiella*, *Pseudomonas*, *Salmonella*, and *Streptococcus* indicates that airport wastewater contains microbial signals consistent with a mixed domestic-like wastewater profile. However, confirmation of bacterial pathogens, toxin genes, or antimicrobial resistance would require complementary bacterial metagenomics, targeted qPCR, culture-based assays, or antimicrobial resistance gene analysis. Therefore, in this study, phage-derived reads are best interpreted as complementary indicators of fecal and microbial community dynamics rather than as direct markers of pathogenic or resistant bacteria. Bacteriophages have been discussed as indicators of fecal pollution and viral pathogen risk, and their relevance as indicators in water and wastewater environments has also been described [46,47].

Taken together, the bacteriophage dataset adds an ecological layer to the interpretation of airport wastewater surveillance. Host-associated phage profiles provided complementary information on the microbial background of airport wastewater and on temporal variation in wastewater inputs, with a persistent *Streptococcus*-associated phage signal and variable contributions from secondary groups such as *Alteromonas*, *Shigella*, *Clostridium*, *Enterobacteria*, and *Stx2*-converting phages. Together with the viral pathogen results, these findings suggest that phage-derived reads can complement human viral detection by providing additional information on the microbial background of airport wastewater, reinforcing its value as a multipathogen and microbial surveillance tool in highly connected environments.

### 3.3. Implications for Airport Wastewater Surveillance

The findings of this study support the use of airport wastewater as a complementary approach for pathogen surveillance in settings characterized by intense population mobility. Airport wastewater surveillance can contribute to the monitoring of SARS-CoV-2 circulation and emerging variants in highly connected environments [2,3,5,6]. Unlike aircraft lavatory wastewater, which may provide more flight-specific information, airport WWTP influent captures a broader operational signal that integrates passengers, workers, commercial areas, sanitary facilities, food services, and maintenance-related activities. Airport and aircraft wastewater may therefore provide complementary but distinct information for pathogen monitoring [10]. Thus, although this integrated profile limits direct attribution to specific traveler groups, it also makes airport wastewater useful for monitoring viral and microbial signals generated by a large and continuously changing population.

The results also highlight the value of moving beyond single-pathogen monitoring. While previous airport wastewater studies have demonstrated the relevance of this approach for specific targets, such as SARS-CoV-2 variants and poliovirus importation, the hybrid-capture target-enriched approach used here enabled the simultaneous detection of multiple human-associated viruses in a single analytical workflow. This broader screening strategy provided a more comprehensive overview of viral signatures potentially circulating in the mixed airport population, including enteric, respiratory, urinary-associated, and arboviral targets. This is particularly relevant in tropical and highly connected regions such as Brazil, where different groups of human viruses may circulate simultaneously. However, as illustrated by the enteric virus findings, interpretation of airport wastewater data should consider the limitations of available clinical surveillance data, especially when etiological confirmation is incomplete or unavailable. Thus, airport wastewater data should be interpreted as complementary to, rather than a replacement for, clinical, epidemiological, and genomic surveillance information.

Some limitations should be considered when translating these findings into surveillance practice. The limited and uneven number of sampling dates restricts stronger conclusions about persistence, recurrence, or seasonality. The seasonal distribution of the samples should also be considered when interpreting the results. Most samples were collected during the austral summer or adjacent warm/rainy-season months, with additional samples collected during autumn and at the beginning of the dry/winter period. Therefore, the dataset does not provide balanced seasonal coverage, and no complete winter sampling period was available. This limitation restricts conclusions about seasonal variation in viral persistence, circulation, and environmental detection in airport wastewater. Future studies should include more frequent and seasonally balanced sampling to better assess temporal and seasonal patterns in viral and bacteriophage-derived signals.

In addition, the hybrid-capture approach used here is useful for detecting selected viral groups, but it does not provide a fully untargeted characterization of the airport wastewater virome. Sequencing-derived abundance estimates may be affected by experimental and analytical biases; therefore, read-based abundance values in this study should be interpreted cautiously because they may be influenced by sample concentration, nucleic acid recovery, target enrichment efficiency, sequencing depth, and bioinformatic classification [39]. Finally, detection of viral nucleic acid does not imply infectivity, and airport wastewater signals are better interpreted as complementary indicators of pathogen circulation trends over time rather than as direct estimates of disease incidence. Future airport surveillance programs would benefit from more frequent sampling, standardized concentrations and sequencing protocols, integration with quantitative assays, and comparisons between airport WWTP influent and aircraft wastewater to better distinguish local operational signals from travel-associated signals.

This study was based exclusively on wastewater samples, and no complementary air or high-touch surface samples were collected at the airport. Therefore, the wastewater data could not be directly compared with other environmental matrices from the same setting. Future airport surveillance studies should consider integrating wastewater, air, and surface sampling to better characterize microbial presence, persistence, and potential exposure pathways in high-traffic built environments.

## 4. Conclusions

In conclusion, this study shows that wastewater from one of Brazil’s largest and most connected airports can provide highly informative signals for pathogen surveillance in environments characterized by intense population mobility. By applying a hybrid-capture target-enriched metagenomic approach, we identified a broad diversity of human-associated viruses and bacteriophage-derived host signatures, including recurrently detected enteric and urinary-associated viruses, as well as the simultaneous detection of SARS-CoV-2 and dengue virus type 1. These findings highlight the capacity of airport wastewater to capture pathogens with distinct transmission dynamics and public health relevance. Importantly, the integration of bacteriophage-associated profiles alongside viral detection represents a novel contribution that expands the ecological and epidemiological interpretation of wastewater metagenomics beyond conventional pathogen monitoring. As one of the first comprehensive viromic studies of airport wastewater in Brazil and among the few conducted in South America, this work highlights the potential of major airports as strategic nodes for environmental genomic surveillance and early detection of emerging and re-emerging infectious diseases. While broader longitudinal sampling and integration with epidemiological data are required to strengthen these observations, the findings provide a robust foundation for incorporating airport wastewater surveillance into routine public health monitoring frameworks in highly connected transit environments.

## Data Availability

The sequences generated in this study were deposited in GenBank (https://www.ncbi.nlm.nih.gov/bioproject/PRJNA1328205, accessed on 27 May 2026) under the sample codes SAMN42174356 to SAMN42174437. Other results are original contributions and are included in the article. Further inquiries can be directed to the corresponding authors.

## Supplementary Materials

The following supporting information can be downloaded at: https://www.mdpi.com/article/10.3390/microorganisms14071402/s1, Table S1: Long-term physicochemical characterization of airport wastewater influent reported by Passos et al. [27]; Table S2: Sequencing depth, quality-control retention, viral enrichment filtering, contig mapping, and viral read assignment for VSP-enriched libraries; Table S3: List of bacteriophage taxa detected in airport wastewater samples and classification according to annotated bacterial host-associated phage groups.

## Abbreviations

The following abbreviations are used in this manuscript:

WBE	Wastewater-Based Epidemiology
WWTP	Wastewater Treatment Plant
NGS	Next-Generation Sequencing
VSP	Viral Surveillance Panel
DNA	Deoxyribonucleic Acid
RNA	Ribonucleic Acid
SARS-CoV-2	Severe Acute Respiratory Syndrome Coronavirus 2
COVID-19	Coronavirus Disease 2019
ND	Not Detected
qPCR	Quantitative Polymerase Chain Reaction
FastQC	Fast Quality Control
SPAdes	St. Petersburg Genome Assembler
BCFTools	Binary Call Format Tools
GATK	Genome Analysis Toolkit
DIAMOND	DIAMOND sequence aligner
Kraken	Kraken taxonomic classification tool
